# A Vision for Incorporating Environmental Effects into Nitrogen Management Decision Support Tools for U.S. Maize Production

**DOI:** 10.3389/fpls.2017.01270

**Published:** 2017-07-28

**Authors:** Kamaljit Banger, Mingwei Yuan, Junming Wang, Emerson D. Nafziger, Cameron M. Pittelkow

**Affiliations:** ^1^Department of Crop Sciences, University of Illinois at Urbana–Champaign, Champaign IL, United States; ^2^Climate and Atmospheric Science Section, Illinois State Water Survey of the Prairie Research Institute, University of Illinois at Urbana–Champaign Champaign, IL, United States

**Keywords:** crop models, corn, in-season nitrogen management, leaching, nutrient recommendation

## Abstract

Meeting crop nitrogen (N) demand while minimizing N losses to the environment has proven difficult despite significant field research and modeling efforts. To improve N management, several real-time N management tools have been developed with a primary focus on enhancing crop production. However, no coordinated effort exists to simultaneously address sustainability concerns related to N losses at field- and regional-scales. In this perspective, we highlight the opportunity for incorporating environmental effects into N management decision support tools for United States maize production systems by integrating publicly available crop models with grower-entered management information and gridded soil and climate data in a geospatial framework specifically designed to quantify environmental and crop production tradeoffs. To facilitate advances in this area, we assess the capability of existing crop models to provide in-season N recommendations while estimating N leaching and nitrous oxide emissions, discuss several considerations for initial framework development, and highlight important challenges related to improving the accuracy of crop model predictions. Such a framework would benefit the development of regional sustainable intensification strategies by enabling the identification of N loss hotspots which could be used to implement spatially explicit mitigation efforts in relation to current environmental quality goals and real-time weather conditions. Nevertheless, we argue that this long-term vision can only be realized by leveraging a variety of existing research efforts to overcome challenges related to improving model structure, accessing field data to enhance model performance, and addressing the numerous social difficulties in delivery and adoption of such tool by stakeholders.

## Introduction

Managing nitrogen (N) on over 130 million ha of cropland is critical for sustainable food production due to the large impact of N fertilizer on farm profits and environmental health (Anderson et al., 2008; Howarth, 2008). In Corn Belt of the United States, only 37 ± 30% of applied N is utilized by the crop (Cassman et al., 2002), with the remaining portion being susceptible to environmental losses. Excessive N leaching from corn fields is a leading source for degradation of water resources (Goolsby and Battaglin, 2001; Ewing and Runck, 2015) while N fertilizer inputs are also linked to increased nitrous oxide (N_2_O) emissions (USEPA, 2014). Therefore, farmers are increasingly faced with the challenge of increasing yields and maximizing farm profits while minimizing negative environmental tradeoffs to meet social demands being placed on agriculture.

Weather patterns in a growing season are a primary factor controlling crop uptake and environmental N losses (Randall and Mulla, 2001). Christianson and Harmel (2015) recently evaluated 40 years of research and found strong relationships between precipitation and N leaching losses, as well as higher crop yields in years with adequate precipitation. In contrast, drought conditions can reduce plant growth and N transport to plant roots, resulting in low N recovery efficiency in dry years (Kim et al., 2008). Interestingly, these weather effects are modified by soil properties and agronomic management, which often result in the development of hotspots of relatively higher N loss (Sogbedji et al., 2001; Kurunc et al., 2011; Tremblay et al., 2012). Thus, it is critical to develop decision support tools that can predict crop yields, soil N supply, and environmental N loss to assist farmers in optimizing N management in United States corn production systems. With the aim of adjusting site-specific N management decisions in the growing season, several existing approaches include soil nitrate tests, sensor based tools, and crop-based diagnostics (Scharf, 2015). Soil nitrate tests are conducted to quantify residual N availability from previous crop as well as to estimate soils N supplying capacity in the corn growing season (Bundy and Malone, 1988; Blackmer et al., 1989; Magdoff, 1991; Schmitt and Randall, 1994). Sensor-based tools include chlorophyll meters (CM) and remote sensing approaches (Shanahan et al., 2008; Ziadi et al., 2008; Tremblay et al., 2011; Yuan et al., 2016). Yet, CM readings show wide variations with respect to cultivars, growth stages, measurement methods, and agronomic practices (Debaeke et al., 2006, 2012; Ziadi et al., 2008). Another practical problem associated with the use of soil nitrate tests and CMs is the time required to obtain representative samples, particularly for larger farms. Remote sensing techniques including hyperspectral imagery can be powerful tools to assess chlorophyll content, biomass, and other biochemical and biophysical properties (Yuan et al., 2016). However, remote sensing methods have limitations resulting in low adoption by farmers including the high cost of sensors and the degree of computer and geospatial skills required to process grid based data, and the need for continuous calibration against soil tests.

To overcome these limitations, a number of site-specific fertilizer recommendation tools based on crop models have recently been developed. For example, Adapt-N which was developed by Cornell University and later commercialized by Agronomic Technology Corporation, is a tool which integrates daily weather, soils data, and field-specific agronomic management information to estimate N recommendations for corn production (van Es et al., 2002). Recently, in 113 on-farm strip trials in Iowa and New York, Adapt-N was found to reduce N rates and N losses by 34 and 38%, respectively, relative to farmers’ practices with no significant difference in yield (Sela et al., 2017). In similar lines, The Climate Corporation has developed FieldView, an online tool capable of providing N recommendations for corn farmers. To our knowledge, the effectiveness of FieldView in maintaining crop yield or reducing environmental N losses has not been assessed. The release of these products represents a breakthrough for this field, with potentially large benefits for crop production.

Despite these promising advances, in this Perspective paper we argue that if these new technologies are to have broader sustainability impacts, several key issues need to be addressed. First, the underlying source codes for both Adapt-N and FieldView are not available in the public domain, which hinders other researchers from improving and integrating new mechanisms associated with soil N supply, crop growth, and environmental loss. For instance, Moore et al. (2014) suggested that quality assurance processes, documentation procedures, and access to model source codes are important aspects while selecting crops models for assessing proposed greenhouse gas abatement methodologies in Australian agriculture. Moreover, previous research has suggested that there are number of non-technological factors such as broad social learning for participatory development of extension specialists, farmers, and scientists to ensure effective adoption of such decision support tools by farmers (Jakku and Thorburn, 2010). Therefore, to enhance the engagement of the broader research community in the development and robust validation of such tools, it is preferable that publicly available models are used rather than commercial products. Second, when making N management decisions, farmers are understandably driven by economic considerations, thus current tools are focused on increasing soil N supply and yields with little emphasis on environmental effects. To encourage use by multiple stakeholders, it is advisable that N loss estimates are converted into actionable knowledge for farmers, for example by providing an interpretation of values with respect to established targets for a farm or region (Ledgard et al., 1999; Cox et al., 2008; Wheeler et al., 2008). Third, and perhaps most importantly, no coordinated effort exists to leverage field-scale N loss estimates to inform the development of regional sustainable intensification strategies. If such tools were to be adopted by farmers at a sufficient scale, information that is not presently available would become available to support novel geospatial assessments of tradeoffs between crop yields and N losses, greatly improving our ability to develop targeted mitigation efforts.

Here we address three areas requiring further attention in order for N management decision support tools to systematically improve environmental sustainability outcomes: the suitability of current, publicly available crop models, initial considerations in geospatial framework development, and important challenges related to model improvements.

## Turning Available Crop Models Into Real-Time N Management Tools

We reviewed the literature to assess the capability of existing publicly available crop models to serve as in-season N management tools for farmers. A total of 12 crop models were assessed according to their current ability to (1) incorporate real-time weather data and provide in-season N recommendations, and (2) simultaneously estimate crop yields and negative environmental tradeoffs related to N management (**Table 1**).

**Table 1:** Status of the current public available crop models to estimate crop-growth and environmental tradeoffs.

Model	Spatial scale	Crop growth	Cultivars within a crop	Environmental tradeoffs				Ability to predict both crop yield and environmental loss?
				Denitrification	N_2_O emissions	Volatilization	Leaching	
APSIM	Point	+	+	+	+	-	+	+
Cropsyst	Point	+	+	+	+	+	+	+
DAISY	Point	+	-	+	-	+	+	+
DAYCENT	Point	+	-	+	+	+	+	+
DNDC	Point and regional	+	-	+	+	+	+	+
DSSAT	Point	+	+	+	-	+	+	+
EPIC	Watershed	+	-	+	-	+	+	-
Maize-N	Point	+	-	-	-	+	-	-
NCSOIL	Point	-	-	-	-	-	-	-
QUEFTS	Point	+	+	-	-	+	-	-
RZWQM	Point	+	+	+	-	+	+	+
SoilN	Point	-	-	-	-	+	+	-


Several existing crop models that are capable of simulating both crop growth and soil N transformations could potentially be used to provide N recommendations in the growing season. However, only Maize-N and QUEFTS are currently used for in-season N recommendations (Janssen et al., 1990; Setiyono et al., 2011). Maize-N combines the Hybrid-Maize model for estimating yield (Yang et al., 2004) with a soil organic matter (SOM) mineralization scheme (Yang and Janssen, 2000) and empirical method for predicting the response of maize yield to N uptake (Setiyono et al., 2011). QUEFTS was primarily developed for tropical conditions with limited application in the United States Midwest. The model recommendations are based on soil fertility status and economic profitability while fewer details are provided on mechanisms controlling the fate of soil N within a growing season.

When models were assessed against the second selection criteria, seven had the ability to simultaneously simulate both grain yield and environmental loss in response to N fertilizer use (Li et al., 1992; Abrahamsen and Hansen, 2000; Jones et al., 2003; Stockle et al., 2003; Xiong et al., 2008; Holzworth et al., 2014). Four models (Maize-N, NCSOIL, QUEFTS, and SoilN) do not incorporate algorithms for estimating denitrification and leaching losses of fertilizer applied N (Molina et al., 1983; Bergstrom et al., 1991; Hanson et al., 1998; Wang et al., 2012). The EPIC model, which is primarily designed to predict the effects of soil erosion on crop productivity, can simulate leaching but not denitrification losses (Wang et al., 2012). As no publicly available models met both criteria, models that estimated environmental tradeoffs are considered here to be the most promising candidates for tool development, assuming they could be integrated with real-time weather as discussed below. Two critical points not considered in this review require further attention. First, important differences may exist regarding the relative capability of these models in predicting N losses vs. crop growth processes and yield (Bassu et al., 2014; Muller et al., 2017). To serve as an indication of which models have a stronger focus on simulating crop growth processes and yield, **Table 1** includes a column noting whether individual cultivars can be specified as an input. Similarly, there is an important difference between whether a model is capable of predicting an output (such as crop yield or N leaching losses) and how extensively the model has been evaluated in predicting an output under a variety of conditions. These aspects would need to be evaluated prior to model selection for geospatial tool development.

**FIGURE 1:**
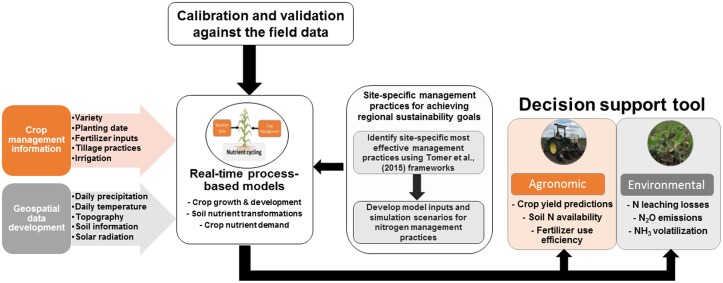
Geospatial framework vision for incorporating environmental effects into N management decision support tools. In the first step, publicly available crop models are integrated with grower-entered management information and gridded soil and climate data to estimate crop growth and soil nutrient transformation processes and quantify sustainability and crop production tradeoffs. To identify site-specific nitrogen management practices for achieving regional sustainability goals, additional attributes to consider in geospatial model development are (1) the need to identify the most effective nitrogen management practices for reducing N losses, for example using watershed-scale frameworks developed by Tomer et al. (2013), and (2) the need for model outputs to be transformed into user-friendly recommendations related to current environmental quality goals such as N leaching and soil N_2_O emissions per unit area and yield.

With a long-term vision of creating in-season N management decision support tools, we now discuss several important attributes to consider for geospatial framework development for corn production systems of the United States (**Figure 1**). The aim of our proposed tool would be to estimate crop yield and environmental loss in the growing season and identify site-specific actions for farmers in the regional sustainability context (Moore et al., 2014). The first step would be designing a user interface by integrating the selected crop model with daily weather, site-specific soils, information and hydrological data from United States Geological Survey (USGS). For the models reviewed above, the majority of previous modeling applications have been conducted retrospectively at individual sites. Since we are unaware of any publicly available model-based framework linking weather, soils, and farmer management to estimate N losses in real-time in United States maize production systems, a real-time modeling interface would represent an important technological advance in this area, with farmers being able to provide minimal inputs including geographical location and relevant crop management practices, while the user interface would automatically incorporate other necessary input data from public resources (Melkonian et al., 2008).

A second consideration would be to transform model outputs into user-friendly sustainability indices for minimizing N leaching and N_2_O emissions associated with N management for corn production systems of the United States. Currently, public and private models do not provide a context for interpreting the magnitude of N loss estimates, thus an opportunity exists to assess whether estimates require action in relation to regional environmental quality goals. For example, if tool developments were focused on N loss to waterbodies in the United States Midwest, watershed-specific numeric nutrient criteria (if available) or regional targets set by United States Environmental Protection Agency (USEPA) could act as thresholds for maximum N losses from farmland (INLRS, 2016). The geospatial framework should be designed to account for farm location in addition to management, as some farms contribute disproportionately higher N loads to waterbodies depending on their geographical location, stream network, and presence of riparian buffer zones (Batie, 1985). It has also been shown that the effectiveness of various practices to control N pollution can vary widely across different geographical locations in a watershed (Arbuckle, 2013). Recently, Tomer et al. (2013) developed a rigorous framework for identifying the most effective site-specific practices for reducing nutrient losses within watersheds at Hydrological Unit Codes (HUC-12). This method or similar approaches could be adapted to serve as a foundation for prioritizing the effectiveness of practices in future tools, recognizing that the resolution of analysis would need to be adjusted to match the goals of the tool (in this case a finer resolution would be needed to estimate farm-level N pollution). Regarding air quality impacts, though several models can predict denitrification loss, only four models (APSIM, Cropsyst, DAYCENT, DNDC) have algorithms to estimate N_2_O emissions (**Table 1**). As a first step, mechanisms for separating N_2_O emissions from denitrification loss should be incorporated and robustly tested against field datasets for these models. Unlike N leaching loss, less information is available to relate N_2_O emission estimates to regional environmental quality targets. As a starting point, crop-specific emissions factors would likely need to act as a reference for farmers, with model-estimated N_2_O emissions being compared to crop-specific emission factors available from public databases such as IPCC or the United States EPA (IPCC, 2006). Given the importance of agricultural N_2_O emissions for climate change, in the longer-term it would be desirable for the proposed N management tool to provide options regarding the effectiveness of formulations, timings, rate, and slow release fertilizers on N_2_O emissions and crop yields. Similarly, various efforts are underway to establish better region- and crop-specific N_2_O mitigation targets (Decock et al., 2015), and these values could be incorporated as they become available.

A third consideration would be to aggregate simulations for field-level crop production and environmental outcomes to larger spatial domains that have been determined to be important for policy-making and strategic investments in agriculture (Grassini et al., 2017). Assuming sufficient farmer participation and adequate spatial coverage, a novel outcome of this framework would be a database containing real-time, spatially explicit estimates of crop growth, nutrient uptake, N leaching, and N_2_O emissions for a given region. While watershed- or ecosystem-scale models have been used in this regard, they often rely on assumptions of uniform crop management within a watershed, thus a strength of the proposed framework would be high-resolution N loss simulations based on accurate crop management information. This type of geospatial information would help advance sustainable intensification planning, which is in part limited due to poor data availability, preventing assessments of how N management simultaneously impacts yield and environmental outcomes. Real-time simulations would also allow the database to be used for identifying N loss hotspots and developing targeted mitigation strategies within a season. Spatial patterns of N loss vary annually, largely according to precipitation. Morever, understanding these patterns will become increasingly important under climate change in the future, as more variable and severe precipitation events are expected.

## Challenges

We recognize there are a number of major limitations that need to be addressed before N recommendation tools can be developed and employed with an acceptable level of confidence (Asseng et al., 2013; Wallach and Thorburn, 2014). Current models may lack accurate mechanistic processes and model parameters for predicting environmental N losses and crop yields (Brilli et al., 2017). For example, an important process regulating soil N availability is SOM mineralization which is highly uncertain in existing models (Benbi and Richter, 2002). Thus, a major challenge is to improve SOM mineralization, with one option being robust model validations of soil respiration in response to environmental factors using field data (Todd-Brown et al., 2013; Tian et al., 2015) A second challenge would be to improve crop growth and development processes (Asseng et al., 2013), particularly the incorporation of cultivars (Archontoulis et al., 2014). In a multi-model intercomparison study, Asseng et al. (2013) described that future climate change impacts on crop yield are highly uncertain due to differences in model structure and parameter values. These specific examples illustrate there is substantial uncertainty in modeling crop and soil processes, particularly under changing environmental conditions and continuously evolving crop management practices. Likewise, when model predictions are scaled up to address regional sustainability concerns, the associated uncertainty needs to be considered (Huffman et al., 2015). A thorough review of modeling challenges is outside the scope of this article, but the reader is referred to several recent reviews highlighting these uncertainties (Benbi and Richter, 2002; Asseng et al., 2013; Vereecken et al., 2016; Brilli et al., 2017).

Ensuring the continuous availability of field data to support model improvements is itself a major challenge. To overcome this limitation, a synchronized research network would be necessary to coordinate different research programs and obtain adequate field data for continuous calibration. We propose that the core of this research network would be field experiments at agricultural universities, which are often designed to investigate the impacts of agronomic management on crop productivity but do not typically feed into modeling efforts. For the development of high resolution historical and current weather datasets, public sector partners such as the United States Department of Agriculture (USDA) Climate Hubs and the Soil Climate Analysis Network (SCAN) would be valuable partners^1^. In the Corn Belt of the United States, an extensive on-farm field research network such as N-Watch would be critical for development of decision support tools. The N-Watch program was started by Illinois Council on Best Management Practices analyze soil samples from farmers’ fields for soil nitrate and ammonium in the corn growing season^2^. The dataset generated by such programs would be an important asset for calibrating model parameters against a wide variety of field data under a range of soil and climate conditions. We argue that the long-term vision proposed in this paper can only be realized by leveraging a variety of existing research efforts, particularly public-private partnerships which have made significant strides in this area.

Accompanying the challenge on the technology side, another major question is how to promote the delivery and adoption of N management tools among farmers. Farmer’s decisions are constrained by the need to maintain farm profits, and what may be considered improved N management practices such as adjusting the formulation, time, rate, and placement of N fertilizers are voluntarily adopted in the Corn Belt of the United States (Reimer et al., 2017). Previous research has suggested that a focus on farmer education and social learning for participatory development is needed for ensuring effective delivery and adoption of decision support tools (Matthews et al., 2008; Jakku and Thorburn, 2010; Hochman and Carberry, 2011). Therefore, major emphasis would need to be placed on addressing the social difficulties associated with the adoption of new N management tools, for example by identifying several management options available to farmers in order to increase awareness while also providing flexibility in adopting the most effective practices to reduce environmental loss. Similarly, regardless of the target region for tool development, a critical role of N management tools will be their ability to maintain or enhance farm profitability while meeting N loss reduction goals.

## Author Contributions

KB and CP developed the conceptual framework and drafted the manuscript. MY assisted in reviewing existing in-season nutrient management tools. JW and EN assisted in revising the manuscript. All authors have read and approved the final manuscript.

## Disclaimer

Opinions expressed are those of the authors and not necessarily those of the Illinois State Water Survey, the Prairie Research Institute, or the University of Illinois.

## Conflict of Interest Statement

The authors declare that the research was conducted in the absence of any commercial or financial relationships that could be construed as a potential conflict of interest.

## Footnotes

1 https://www.climatehubs.oce.usda.gov/midwest

2 http://www.illinoiscbmp.org/Nitrogen-Management/N-Watch
